# Summer Residence for the Consumptive

**Published:** 1834

**Authors:** K. McKinney

**Affiliations:** Princeton, N. J.


### VIII.—Summer Residence for the Consumptive.

*Princeton, N. J.* 23d *May,* 1834.

Dr. Drake,

Dear Sir,

Since I enjoyed the honor of your public and private instruction in 1826–7, I have indulged my favorite propensity of travelling; always uniting my excursions as much as possible with my medical pursuits. In 1829 I visited Yucatan, one of the United Mexican States, where I have resided nearly ever since. Campeche (my residence) has a clear, dry and salubrious atmosphere, and is remarkably healthy, notwithstanding the culpable neglect of the police. There is no winter, and it is very seldom the thermometer falls below 64°. There are no marshes in the vicinity, and atmospheric vicissitudes are gradual and not very considerable: hence febrile and catarrhal affections are rare, unless brought on by undue exposure to rain or the intense rays of a tropical sun.

These circumstances induce me to think that this would be a valuable winter residence to those of our unfortunate countrymen predisposed to, or laboring under phthisical affections, and, in fact, persons of delicate health from whatever cause. Living is cheap, and the market pretty well supplied, and the climate milder than that of the Island of Cuba or the South of Europe, and the expenses are not the third part as much as in the former places. A difficulty, however, exists: The language, religion and manners of these people, are different from ours, which prevents those friendly offices which ought to exist between the host and his guest. It is hence an inquiry of importance, whether a suitable substitute can be found within the limits of our own country. Is there any part of these United States combining the mild and salubrious climate of the tropics, with the beneficient influence of our free institutions?

In my return from Campeche, I visited Key-West and Cape Florida, on an exploring tour; I was careful to inform myself relative to the soil and productions; but especially relative to the climate. From all I can learn, Cape Florida enjoys a very mild and grateful temperature during the whole year. Frost is entirely unknown. The most delicate plants vegetate perfectly well in the midst of January. I saw at Cape Florida ripe corn, corn silking, and corn just sprouting through the ground, in the month of February. The plantain, banana, and papaya, which require a year to ripen, grow there to great perfection. One might suppose, from its latitude, that Cape Florida would be colder than the Havana; but when it is recollected that there are no elevated lands in the vicinity, and that the prevailing north winds sweep for hundreds of miles the surface of the heated Gulf stream, it is readily seen why the atmosphere is as mild or even milder than that of the great Emporium of Cuba. Cape Florida will ultimately be the Italy of our country. Its warm and salubrious atmosphere far excels the damp and chilling breezes of St. Augustine and the south-western states: It far surpasses the farfamed Languedoe and Leghorn: Its waters are clear, pelucid and wholesome: Its rivers abound with fish and fowl, and its wôods in game: Its trees yield cooling and refreshing fruits; and even the piny barrens, so sterile elsewhere, yield a root in great abundance (Compti or Florida Sagoplast) from which is extracted a beautiful and nutritious aliment, equal to the best Indian arrowroot. The whole abound in good pasturage, well situated for the growth of live stock, with a very trifling expense. Difficulties, as yet, attend the settling of this country. The Spanish Grants are not yet adjusted, and the Government lands have not been surveyed. Hence, most of the inhabitants are squatters who gain a precarious livelihood by fishing and hunting. Mr. Fitzpatrick, however, has purchased a large tract of land at the mouth of the Miami river, where he is actually engaged in opening a farm, preparatory to erecting a large edifice for the reception of invalids, and as Mr. F. possesses ample means it is to be hoped that much time will not elapse before he shall have completed his undertaking.

The survey and sale of these lands ought to occupy the attention of Congress at an early period: That suitable residences and provison may be made for all who from misfortune or from choice may wish to visit these sunny regions. Those who have lived long in that country, represent it as very healthy, fevers being scarcely known.

Many medicinal plants are already found, and doubtless more will be discovered, when these interesting regions shall have been sufficiently explored by scientific men. May it not also be converted into a site for the cultivation of medicinal plants and Botanical specimens, without the great inconvenience and expense of green houses? By placing the Cape Florida lands in market, we open a new source of wealth to our citizens, a vast theatre of scientific research to our philosophers, and we secure any asylum for our unfortunate friends, free from the chilling blasts of winter, and the more *chilling indifference* of "strangers in a strange land."

I would solicit your efforts in behalf of this section of our country, that the attention of the profession may be called to a subject in which a large portion of invalids are deeply interested.

Very respectfully, your friend
and former pupil,
K. McKINNEY.

